# Supplementary material for: Obesity Induces an Impaired Placental Antiviral Immune Response in Pregnant Women Infected with Zika Virus
Source: Viruses. 2023 Jan 23;15(2):320. doi: 10.3390/v15020320 (PMC9966111; doi:10.3390/v15020320)
Supplement: Supplementary file 1 [file viruses-15-00320-s001.zip › viruses-2150241-supplementary.pdf]

Table S1. Oligonucleotides used in real-time PCR experiments

Target	Sequences
**TLR3**	F: AGTGCCGTCTATTTGCCACA<br>R: TGATTCTGTTGGATGACTGCT
**TLR7**	F: AATGTCACAGCCGTCCCTAC<br>R: GCGCATCAAAAGCATTTACA
**IRF-3**	F: AGAGGCTCGTGATGGTCAAGGTT<br>R: AGAGTGGGTGGCTGTTGGAAATG
**STING**	F: ATATCTGCGGCTGATCCTGC<br>R: GGTCTGCTGGGGCAGTTTAT
**RIG-I**	F: CTGGACCCTACCTACATCCTG<br>R: GGCATCCAAAAAGCCACGG
**MxA**	F: AAGCTGATCCGCCTCCACTT<br>R: TGCAATGCACCCCTGTATACC
**ISG 15**	F: TGGCGGGCAACGAATT<br>R: GGGTGATCTGCGCCTTCA
**IFN-α**	F: AAATACAGCCCTTGTGCCTGG<br>R: GGTGAGCTGGCATACGAATCA
**IFN-β**	F: CATTACCTGAAGGCCAAGGA<br>R: CCATTGTCCAGTCCCAGAGG
**IFN-λ**	F: CGCCTTGGAAGAGTCACTCA<br>R: GAAGCCTCAGGTCCCAATTC
**YWHAZ**	F: GCCACAATGTTCTTGGCCCATCAT<br>R: TGGTTGGTGACAAGACAGAAGGCT
